# Endoplasmic Reticulum-Based Calcium Dysfunctions in Synucleinopathies

**DOI:** 10.3389/fneur.2021.742625

**Published:** 2021-10-20

**Authors:** Gergo Kovacs, Lasse Reimer, Poul Henning Jensen

**Affiliations:** ^1^Danish Research Institute of Translational Neuroscience – DANDRITE, Aarhus University, Aarhus, Denmark; ^2^Department of Biomedicine, Aarhus University, Aarhus, Denmark

**Keywords:** Parkinson's disease, α-synuclein, calcium, endoplasmic reticulum, SERCA, RyR, IP3R

## Abstract

Neuronal calcium dyshomeostasis has been associated to Parkinson's disease (PD) development based on epidemiological studies on users of calcium channel antagonists and clinical trials are currently conducted exploring the hypothesis of increased calcium influx into neuronal cytosol as basic premise. We reported in 2018 an opposite hypothesis based on the demonstration that α-synuclein aggregates stimulate the endoplasmic reticulum (ER) calcium pump SERCA and demonstrated in cell models the existence of an α-synuclein-aggregate dependent neuronal state wherein cytosolic calcium is decreased due to an increased pumping of calcium into the ER. Inhibiting the SERCA pump protected both neurons and an α-synuclein transgenic *C. elegans* model. This models two cellular states that could contribute to development of PD. First the prolonged state with reduced cytosolic calcium that could deregulate multiple signaling pathways. Second the disease ER state with increased calcium concentration. We will discuss our hypothesis in the light of recent papers. First, a mechanistic study describing how variation in the Inositol-1,4,5-triphosphate (IP3) kinase B (ITPKB) may explain GWAS studies identifying the ITPKB gene as a protective factor toward PD. Here it was demonstrated that how increased ITPKB activity reduces influx of ER calcium to mitochondria via contact between IP_3_-receptors and the mitochondrial calcium uniporter complex in ER-mitochondria contact, known as mitochondria-associated membranes (MAMs). Secondly, it was demonstrated that astrocytes derived from PD patients contain α-synuclein accumulations. A recent study has demonstrated how human astrocytes derived from a few PD patients carrying the LRRK2-2019S mutation express more α-synuclein than control astrocytes, release more calcium from ER upon ryanodine receptor (RyR) stimulation, show changes in ER calcium channels and exhibit a decreased maximal and spare respiration indicating altered mitochondrial function in PD astrocytes. Here, we summarize the previous findings focusing the effect of α-synuclein to SERCA, RyR, IP_3_R, MCU subunits and other MAM-related channels. We also consider how the SOCE-related events could contribute to the development of PD.

## Old and New Hypotheses on Ca^2+^ in Parkinson's Disease and Synucleinopathies

Parkinson's disease (PD) is the second most common neurodegenerative disorder with more than 6 million diagnosed cases, and more than 100 000 deaths/year (1, 2). It belongs to the group of neurodegenerative synucleinopathies, dominated by PD, dementia with Lewy bodies (DLB) and Multiple system atrophy (MSA). These diseases are hallmarked by the presence of inclusions in degenerating brain cells that contain aggregates of the presynaptic protein α-synuclein (α-syn). In PD and DLB inclusions are in neurons whereas they predominantly are located in oligodendrocytes in MSA.

Our insight into the pathophysiology of the synucleinopathies has evolved greatly since α-syn was identified as a presynaptic protein (3) and first associated to neurodegenerative diseases as a component of amyloid plaques in Alzheimer's disease (4). The breakthrough came in 1997 when a missense mutation in *SNCA* (the gene encoding the α-syn protein) was identified as causing autosomal dominant PD (5) and α-syn was identified as an unifying component of Lewy bodies in PD and DLB and in glial cytoplasmic inclusions in MSA (6, 7). Later, several missense α-syn mutations as well as multiplication of the *SNCA* gene has been demonstrated as causing autosomal dominant forms of PD and DLB (8). The progressive nature of the Lewy body pathology was described by Braak that proposed the so-called Braak hypothesis. It postulates pathology to spread from two places in the nervous system: neurons in the olfactory bulb and also neurons of the gut that via the Vagal nerve spread to the brain stem and further progress to the substantia nigra and neocortical areas (9, 10). Recent data suggest an opposite route of spreading also exists, so-called top down PD, from the central nervous system to the peripheral nervous system (11). These routes of spreading are hypothesized to be brought about by a prion-like spreading of α-syn aggregates from degenerating neurons to connected neurons where they seed the aggregation of native α-syn in the healthy recipient neurons.

The loss of the dopamine producing neurons of the substantia nigra pars compacta (SNpc) forms the basis for the motor symptoms that is diagnostic for PD. The demonstration of slow rhythmic calcium (Ca^2+^) oscillation in this population of pacemaking dopaminergic (DA) neurons driven by Ca^2+^ influx through CaV1 Ca^2+^ channels, along with their low Ca^2+^ buffering capacity and increased oxidative stress formed part of the basis for an almost 30-year-old calcium hypothesis (12–14). It gained strong support from epidemiological studies demonstrating a decreased risk for PD in users of L-type Ca^2+^ channel antagonists (15, 16), although the latter study rather interpreted their data as supportive for a symptomatic effect. Influenced by these findings, isradipine, a CaV1.3 channel antagonist with promising pre-clinical neuroprotective effects in some PD animal models (17–19), was administered to patients with early-stage PD for 36 months in a large clinical trial (20). Yet, despite encouraging pre-clinical studies, isradipine failed to slow the clinical progression of early-stage PD patients. This might be explained by insufficient dosing of the drug in the clinical study, but it could also reflect that this calcium hypothesis does not reflect the underlying disease mechanisms driving the development and progression of PD through the nervous system.

**Figure 1:**
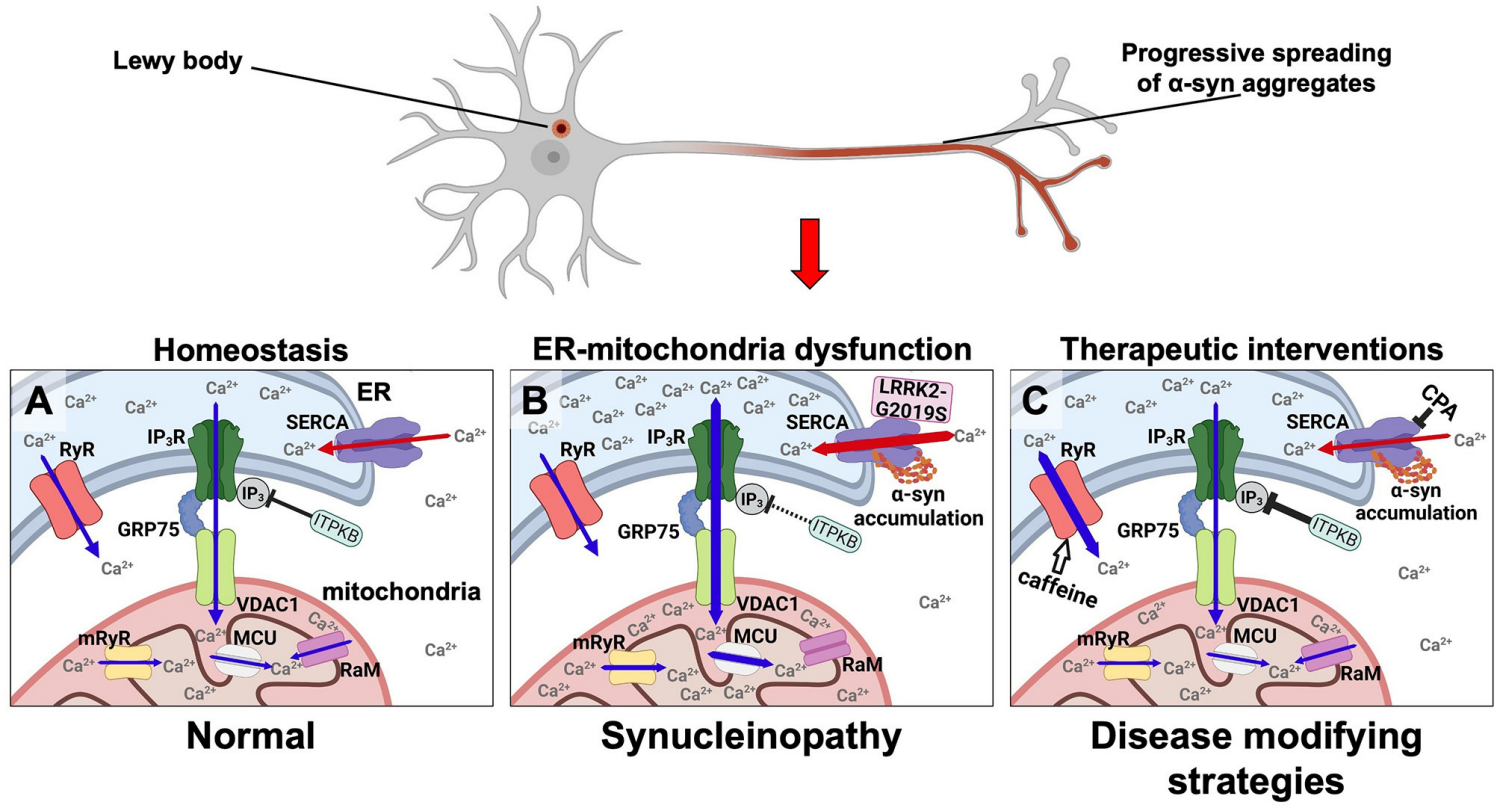
Disease modifying strategies for synucleinopathies by modulating aberrant Ca^2+^ fluxes in ER and mitochondria via targeting the SERCA pump, the RyR, and the ER-mitochondrial transport complex. α-syn is under normal conditions predominantly located in nerve terminals. In synucleinopathies, its initial aggregation is hypothesized to initiate at this site. From here, aggregates are transported retrograde through the axon to the cell body where Lewy bodies can form. **(A)** In healthy neurons the low resting Ca^2+^ level in the cytosol and the high Ca^2+^ levels in the ER and mitochondria are tuned by (1) the SERCA pump loading the ER with Ca^2+^ from the cytosol (red arrows), (2) the release of Ca^2+^ from ER to the cytosol by RyR and IP_3_R (blue arrows), (3) where the latter is part of the multi-molecular Ca^2+^ funnel between ER lumen and the mitochondrial matrix (IP_3_R, GRP75, VDAC1, mRyR, MCU, and RaM). **(B)** In synucleinopathies, the α-syn aggregates activate SERCA causing an increased Ca^2+^ uptake into the ER thereby deregulating ER functions and increasing the Ca^2+^ flux through the IP_3_R-GRP75-VDAC1 complex and MCU into mitochondria. These increased Ca^2+^ fluxes into ER and mitochondria along with a decreased cytosolic Ca^2+^ concentration result in a reduced functionality and viability of neurons in synucleinopathies. The same pathways can be activated by mutant LRRK2-G2019S that activates SERCA and by loss-of-function genetic risk variants in the ITPKB kinase that increases the IP_3_-dependent Ca^2+^ flux from ER to mitochondria. **(C)** These abnormal Ca^2+^ fluxes can be counteracted as a disease modifying strategy in synucleinopathies. The α-syn activated SERCA can be inhibited e.g., by CPA. The Ca^2+^ flux from ER to mitochondria can for example be attenuated by stimulating ITPKB or inhibiting the IP_4_ phosphatase. The Ca^2+^ flux from ER into the cytosol can be stimulated by caffeine (white arrow) or other RyR activators.

In 2018 we offered an alternative calcium hypothesis founded on progressive α-syn aggregate toxicity in neurons as a basis for the progression of PD (Figures 1A,B). It was based on our demonstration of a biphasic cytosolic Ca^2+^ response caused by α-syn aggregates that bind and stimulate the endoplasmic reticulum (ER) Ca^2+^ pump SERCA thereby enhancing its pumping of Ca^2+^ from the cytosol into the ER. This activation causes an initial prolonged phase characterized by a decrease in cytosolic Ca^2+^ along with an increased Ca^2+^ loading of the ER that is followed by a second degenerative phase with increased cytosolic Ca^2+^ prior to cell death (21). Both phases could be counteracted by treating the neurons and a transgenic *C. elegans* model with low doses of the SERCA inhibitor cyclopiazonic acid (CPA) (Figure 1C). The prodegenerative signaling pathways activated in the early and in the late phases are yet to be discovered. Given that PD is a slowly progressive disease hypothesized to be driven by a spreading of α-syn aggregates through the nervous system, we envision that at any given time there will be some neurons in the frontier of spreading that are affected by the first low Ca^2+^ phase and other areas affected for longer time by the late high Ca^2+^ phase. Treatment of the SERCA activation thus holds potential of reducing the dysfunction of the neurons, e.g., symptomatology, but also modification of the progression of the disease.

In the current review we will primarily focus on Ca^2+^ regulatory molecules in the ER in relation to synucleinopathy-related pathology and discuss how our SERCA activation calcium hypothesis align with recent publications implicating mitochondria in this pathway.

## Organelles, Ca^2+^ Fluxes and their Interplay in PD

Neuronal Ca^2+^ influx is a vital component in the process of neurotransmission from the regulation of dendritic responses to neurotransmitter binding, propagation of action potential along axons and transmitter release from nerve terminals; and also serves as a second messenger in signaling pathways regulating multiple processes, including metabolism, protein phosphorylation, exocytosis, gene transcription and programmed cell death (22, 23). Each of these pathways can affect neuronal function and neurodegenerative processes, thus Ca^2+^ dyshomeostasis is proposed to play a vital part in complex brain processes like age-dependent cognitive decline and development of e.g., schizophrenia (24, 25). Maintenance of specific subcellular Ca^2+^ levels is therefore a prerequisite for a plethora of cellular processes and their integrated support of the mind. Neuronal Ca^2+^ signaling is highly advanced and mediated by various ion channels, exchangers, and pumps situated on the plasma membrane (PM) and membranes of different organelles such as ER, mitochondria, lysosomes, Golgi apparatus and others (23). In the following section we will briefly outline the role of the ER and mitochondria representing two central organelles in Ca^2+^ transport, buffering and whose proper function depend on their luminal Ca^2+^ levels (Figures 1A,B). This will include description of their interphases and the ER interphase with PM, and a discussion on how dysregulation of specific Ca^2+^ pumps and channels in these organelles might play a role in the etiology and pathology of PD and other synucleinopathies.

### Endoplasmic Reticulum (ER)

ER forms an interconnected network of tubules throughout the neuron from dendrites to nerve terminals (26). It is in close contact with many other intracellular organelles and their communication has essential functions in maintaining Ca^2+^ homeostasis during physiology processes and disturbance herein holds potential for facilitating psychiatric symptoms and neurodegenerative processes (27). While the resting cytosolic Ca^2+^ concentration in neurons is in the nanomolar range (28), in the ER and in the extracellular compartments it is in the millimolar range (29, 30). To maintain such steep gradients, active transporters are necessary, and in the case of ER, the Sarco/endoplasmic reticulum Ca^2+^ ATPase (SERCA) carries the load. Together with the PM Ca^2+^ ATPase (PMCA), SERCA, and Na^+^/Ca^2+^ exchangers (NCX) contribute to maintain a low resting cytosolic Ca^2+^ concentrations in neurons (31).

#### The Active Player for Ca^2+^ Uptake–The SERCA Pump

SERCA plays a crucial role in maintaining ~150 nM concentration of Ca^2+^ in the cytosol and do this by pumping the ions against a ~1 mM Ca^2+^ gradient into the ER at the expense of ATP hydrolysis. SERCA pumps has a quite complex biology (32): they are expressed ubiquitously from three genes, *ATP2A1, ATP2A2*, and *ATP2A3*; and taking alternative splicing into account, 13 different isoforms exist (33, 34). The molecular weight of SERCA is usually referred as 110 kDa however the different isoforms have different molecular weights between 95 and 115 kDa (35, 36). The different SERCA isoforms are expressed in a developmental and tissue specific manner with the SERCA2B and SERCA3 being dominant in the brain (37).

We have shown that α-syn aggregates bind to and stimulate SERCA resulting in an increased Ca^2+^-load in the ER-lumen and a reduced cytosolic Ca^2+^ level. Following a prolonged phase with reduced cytosolic Ca^2+^, yet unknown homeostatic mechanisms deteriorate resulting in increased cytosolic Ca^2+^ and cell death (21). These two cellular states may contribute to development and progression of PD where the first phase with reduced cytosolic Ca^2+^ could deregulate multiple signaling pathways affecting the circuitry functions of neurons and thus contribute to a range of neurological and psychiatric symptoms. The second phase with increased Ca^2+^ concentration will then initiate the irreversible phase with enhanced neuron loss. The latter phase may resemble aspects of the “old” calcium hypothesis. We demonstrated the specific SERCA inhibitor CPA was able to counteract both phases of Ca^2+^ deregulation caused by α-syn aggregate stress and as a result increased the viability of primary neuron models and protected against cell loss in a *C. elegans* model (21) (Figure 1C).

Astrocytes has been linked to α-syn pathology after Braak reported that astrocytes in PD patients of advanced stages were immunoreactive for α-syn (38) and such astrocytic α-syn causes proinflammatory stress from the astrocytes (39). Genetic and molecular evidence has recently linked the LRRK2-G2019S mutant, which is the most common dominant cause of familial PD (40, 41), to SERCA, ER and mitochondrial stress in astrocytes (42, 43).

#### Ca^2+^ Release Channels–IP_3_R and RyR

The release of Ca^2+^ ions from the ER in neurons is mediated through two distinct receptor channels, namely the Inositol-1,4,5-trisphosphate receptors (IP_3_Rs) and Ryanodine receptors (RyRs) (44, 45). Contrary to the RyRs, which only are expressed in excitable cells like neurons and muscle cells, the IP_3_Rs show ubiquitous expression patterns.

##### IP_3_R

The IP_3_R is a Ca^2+^ channel located in ER membrane that is activated by binding of Inositol-1,4,5-trisphosphate (IP_3_). The IP_3_ is generated by phospholipase C action on the membrane phospholipid Phosphatidylinositol 4,5-bisphosphate (PIP_2_) in a process often associated to G-protein coupled receptor activation. The IP_3_R is critically involved in the transport of Ca^2+^ from ER to mitochondria through its interaction with the major mitochondrial Ca^2+^ transport channel located in the outer mitochondrial membrane, called Voltage-dependent anion channel 1 (VDAC1) in a complex bridged by the chaperone GRP75 (75 kDa Glucose-regulated protein) (46, 47). This interaction occurs at specialized ER-mitochondrial interphases known as mitochondria-associated membranes (MAMs). The tethering complex of IP_3_R-GRP75-VDAC1 allows the efficient diffusion of Ca^2+^ from ER into mitochondria. Other chaperones, like calnexin and calreticulin can also interact with IP_3_R and SERCA2B to regulate mitochondrial Ca^2+^ homeostasis (48). There are 3 known isoforms of IP_3_R encoded by *ITPR1, ITPR2*, and *ITPR3* genes, that are expressed as 312, 308, and 304 kDa protein products, respectively (49, 50). Although all three isoforms are expressed ubiquitously, there is also a heterogeneity in their expression pattern in different tissues and in their function (51).

The activation of IP_3_R is not just regulated by phospholipase C dependent release of IP_3_ but can also be modulated by inactivating IP_3_ via its phosphorylation to IP_4_ by IP_3_ kinases. The IP_3_ kinase B (ITPKB) is such an inactivating kinase (52). It is ubiquitously expressed and it is the most abundantly expressed IP_3_ kinase in the central nervous system across several brain regions related to PD (53). *ITPKB* is a risk gene for sporadic PD (54) and recently, Apicco et al. investigated how modulating ITPKB activity in neurons by genetic and pharmacological methods impacted the accumulation of pS129 labeled α-syn aggregates in human neuron and *in vivo* rodent models (55). They demonstrated that in neurons ITPKB activity negatively regulate the transfer of Ca^2+^ from ER stores to mitochondria. Here the increased Ca^2+^ is associated with functional changes in mitochondria, including increased respiration, ATP production, and the accumulation of reactive oxygen species (ROS). In summary, this molecular study indicated that the genetic PD risk contributed by the *ITPKB* locus is caused by its modulation of the Ca^2+^ flux from ER to mitochondria (55). We find it tempting to speculate that an increased Ca^2+^ load in ER caused by α-syn aggregates activation of SERCA likewise will favor a pathogenic Ca^2+^ flux from ER to mitochondria (Figure 1B).

##### RyR

The RyRs exists in three tissue-specific isoforms encoded by *RyR1, RyR2*, and *RyR3* genes. While the 565 kDa RyR1 is mainly expressed in skeletal muscles and cerebellar Purkinje cells, the 565 kDa RyR2 is the predominant isoform in the brain and heart, and the 552 kDa RyR3 is brain-specific albeit with low expression levels (23). The RyR channels forms by homo-tetramerization, and thereby constitutes the largest known intracellular ion channel with a size that exceeds 2 MDa. Although the role of RyR in the brain is still incompletely understood, several lines of evidence point toward involvement in long term potentiation (LTP) and long term depression (LTD) (56, 57). A recent genome-wide association study (GWAS) suggests a role in PD, where a specific SNP (Single Nucleotide Polymorphism) variant of the *RyR2* gene was associated with a lower cognitive score in PD patients (58).

Structurally, the transmembrane pore of the RyR, in which the Ca^2+^ ions are transported is formed by the C-terminal part of the receptor, while a large cytoplasmic region of the channel is the interaction site of most RyR modulators (23). One modulator of RyR is Ca^2+^ itself, which can act as an activating ligand. This also explains how an increase in cytosolic Ca^2+^ triggers further Ca^2+^ release in a mechanism known as Ca^2+^-induced Ca^2+^ release (CICR). On the other hand, a detrimental decrease in cytosolic Ca^2+^, e.g., due increased activation of SERCA by α-syn aggregates (21), could in theory be aggravated by a decrease in RyR activation due to the reduction of its Ca^2+^ ligand. Interestingly, an increase in cytosolic Ca^2+^ more easily activates the brain-predominant isoforms, RyR2 and RyR3, compared to RyR1 (59) suggesting a prominent role of CICR in brain. Besides Ca^2+^, another cellular activation pathway of RyR via cyclic adenosine diphosphate ribose (cADP-ribose) exists (60). Pharmacologically RyRs can be activated by nanomolar concentrations of the plant alkaloid ryanodine, whereas high concentrations are inhibitory (61). Caffeine is a well-known activator of RyR that sensitize RyR to its activation by cytosolic and luminal Ca^2+^ to facilitate spontaneous Ca^2+^ release (62–65). RyR was also identified in rat heart mitochondria (66), named as mitochondrial Ryanodine receptor (mRyR), where it is located in the inner mitochondrial membrane and partially is responsible for facilitating rapid mitochondrial Ca^2+^ uptake. mRyR has also been demonstrated in mitochondria of primary cultures of rat striatal neurons (67). Using subtype specific antibodies and RyR1 knockout mice tissue it was demonstrated that cardiac mRyR is the RyR1 isoform (68).

According to our calcium hypothesis, which postulates that a normalization of an otherwise decreased cytosolic Ca^2+^ level will alleviate PD-related pathophysiology, a caffeine-induced release of Ca^2+^ from the ER through RyR would be favorable. Interestingly, several epidemiological studies point toward a protective role of caffeine in relation to PD onset [for a review, see (69)]. A clinical study demonstrated improved objective motor measures in PD patients treated with caffeine (70), and another study found an association between coffee consumption and improved mood and cognition in PD patients (71). The short-term nature of the studies may have missed a positive disease modifying potential considering the slowly progressive nature of PD and lacking good biomarkers of disease progression. In line with this, plasma concentrations of caffeine and its metabolites, paraxanthine, theophylline and 1-methylxanthine, was significantly lower in both sporadic PD patients, and PD patients carrying an *LRRK2* mutation, relative to unaffected controls (72). Chronic caffeine treatment attenuated α-syn inclusion formation and reverted autophagic defects caused by intrastriatal injection of preformed α-syn fibrils (PFFs) in an α-syn transgenic mouse model (73) (Figure 1C). Caffeine is a somewhat promiscuous drug with a complex pharmacological profile where antagonism of adenosine receptors is often considered the main pharmacological target, and the current leading hypothesis in the field is that caffeine exerts its protective effects through antagonizing adenosine A_2A_ receptor (69). The adenosine A_2A_ receptor antagonist istradefylline is approved for treatment of PD patients (74) but istradefylline did not, compared to caffeine, protect against the progressive neurodegeneration in our yet unpublished preclinical study using the A53T-α-syn transgenic M83 model (Betzer et al., unpublished). It is thus yet unclear how caffeine mechanistically decreased the progression of α-syn in preclinical models of synucleinopathies [(73) and Betzer et al., unpublished] but could in principle be via activation of RyR thus supporting our calcium hypothesis.

Another protein that may contribute to the interplay between molecules regulating Ca^2+^ in the ER is the Soluble resistance-related Ca^2+^-binding protein (SORCIN) that upon Ca^2+^ binding is able to modulate the function of SERCA and RyR (75).

#### Ca^2+^ Flux Across the Plasma Membrane Into the ER–Store Operated Ca^2+^ Entry (SOCE)

The ER can also form Ca^2+^ conducive membrane interphases with the PM (76) and this interaction is critical for the process of store operated Ca^2+^ entry (SOCE) into the ER and cytosol from the extracellular space with the tethering of the membranes mediated by the proteins ORAI and Stromal interaction molecules (STIMs) (76). SOCE plays an important role in the nerve terminals where α-syn is present in high concentrations (77). Upon Ca^2+^ depletion of ER, Ca^2+^-selective store-operated channels (SOCs) are assembled by interaction between ER Ca^2+^-sensing STIM1/2 proteins and plasma membrane-associated ORAI1/2/3 proteins, which together form the PM Ca^2+^ release-activated Ca^2+^ (CRAC) channels, thereby allowing restoration of ER Ca^2+^ stores by influx from the extracellular space (78). In addition to ORAI proteins, the transient receptor potential canonical channels (TRPC) have also been suggested to participate in CRAC channel formation (78, 79). SERCA participates in termination of SOCE by restoring ER its high Ca^2+^ levels (80).

By inducing Ca^2+^ influx into the cytoplasm and ER as a consequence of ER Ca^2+^ depletion, SOCE plays an important role in maintaining neuronal Ca^2+^ homeostasis, and SOCE has been linked to a multitude of biological processes, including transcription, exocytosis, and metabolism (78). The α-syn aggregate dependent activation of SERCA that increases ER Ca^2+^ levels holds potential for disturbing SOCE (21).

Interestingly, post-mortem SNpc samples from PD patients demonstrated a decreased presence of TRPC1 compared to non-PD patients, which could suggest a downregulation of SOCE (81). Human primary skin fibroblasts derived from patients with either familial PD, caused by mutations in *PLA2G6* gene (PARK14), or idiopathic PD also show impaired SOCE relative to those of control patients (82). Moreover, *Pla2g6* knockout mice displayed autophagic dysfunctions, loss of SNpc DA neurons and an age-dependent motor dysfunction (82).

#### ER Stress Responses

Apart from storing Ca^2+^ and securing a low cytosolic Ca^2+^ level, several functions of the ER is critically dependent on a regulated luminal Ca^2+^ level like folding of secretory proteins and membrane proteins for Golgi, lysosomes and the plasma membrane; and lipid and sterol biogenesis (83). Perturbation of these functions can cause the unfolded protein responses (UPR) (84). UPR can be induced by environmental or genetic insults that result in dysfunctional protein folding in the ER lumen (85, 86). Experimentally UPR has been induced by lowering the ER Ca^2+^ level by the SERCA inhibitor thapsigargin and the inhibitor of protein glycosylation tunicamycin (87, 88). The prototypic stress response aims at restoring the protein folding homeostasis in the ER and is dominated by three transmembrane ER proteins PERK (PKR-like ER kinase), IRE1α (Inositol-requiring transmembrane kinase/endoribonuclease 1α), and ATF6 (Activating transcription factor 6) that all become activated when the ER chaperones BiP and GRP78 (78 kDa Glucose-regulated protein) are released upon their interaction with misfolded proteins (89, 90). α-Syn aggregate stress has been linked to the UPR pathway (91, 92), and salubrinal, an eIF2α phosphatase inhibitor that attenuates ER stress delayed the onset of α-synucleinopathy in A53T transgenic mice (93). α-syn aggregates has been demonstrated to accumulate at ER in sick α-syn transgenic mice and in ER containing microsomes from PD brains (89, 93).

Missense mutations in the lysosomal enzyme glucocerebrosidase are responsible for Gaucher's disease that is a strong risk factor for PD. The pathophysiology for some mutations likely contains a component of ER stress as the RyR blocker Dantrolene was able to alleviate disease in a mouse model of Gaucher's disease (94).

GWAS studies have revealed that genetic risk factors for sporadic PD frequently encode for proteins involved in lysosomal function and autophagy, processes relying on ER function (54). This is for example demonstrated by the generation of the double membrane of autophagosomes that engulf cargo for degradation by macroautophagy. The double membrane is derived from the ER membrane or ER-mitochondrial contact sites (95). Attenuation of the α-syn aggregate activated SERCA pump in neurons decreased their cellular α-syn level suggesting a direct link between the ER dysfunction and α-syn regulating autophagy (21). Inhibition of ER in neurons by knockout of *ATG5* surprisingly revealed that the reticular ER in axons is a primary target for neuronal autophagy and demonstrated the ER resident RyR Ca^2+^ channels as especially sensitive to autophagic dysfunction (96). The changes in ER by autophagy inhibition did change axonal and ER Ca^2+^ levels but did remarkably not elicit the conventional ER response driven by the PERK/IRE1a and ATF6 signaling axis (96) although it affected neuronal transmission as also observed for ER stress (97).

### Ca^2+^ Fluxes From ER to Mitochondria

Although ER is considered the main Ca^2+^ store within cells, mitochondria also play a key function in buffering Ca^2+^ ions. Moreover, Ca^2+^ plays a pivotal role in mitochondria as oxidative phosphorylation requires mitochondrial Ca^2+^ and dysregulation hereof can generate oxidative stress associated with cell death in neurodegenerative diseases (98). Modest accumulation of Ca^2+^ in the mitochondria can lead to decreased respiration, while further increase in Ca^2+^-level results to the degradation of metabolic enzymes through the activation of mitochondrial Calpain 1, and also increases ROS generation by impairing of respiratory enzymes. Oxidative stress, Ca^2+^-overload in mitochondria and ROS-generation are amplifying each other's effect which leads to mitochondrial damage (99, 100). In neurons, increased cytosolic Ca^2+^ can inhibit both anterograde and retrograde transport of mitochondria to axons and dendrites, where mitochondria act as Ca^2+^ buffers to sustain synaptic activity (101).

The Ca^2+^ flow from ER to mitochondria relies on the proximity of their membranes in the MAMs structure (47). Here tethering of IP_3_R to VDAC1 is facilitated by the cytosolic GRP75 thereby creating a funnel facilitating the diffusion of the high concentration of Ca^2+^ in ER into the intermembranous mitochondrial space. The further diffusion into the mitochondrial matrix is facilitated via the mitochondrial Ca^2+^ uniporter (MCU), mRyR and the so-called rapid mode uptake channel (RaM) located in the inner mitochondrial membrane (67, 102, 103). RaM, which exact molecular identity is still unknown, facilitates a rapid mode of mitochondrial Ca^2+^ uptake with faster kinetics than MCU but, interestingly, high extra-mitochondrial Ca^2+^ level has inhibiting effect to RaM (102). The importance of this pathway is highlighted by the PD risk variants of the IP_3_ kinase ITPKB that increases the Ca^2+^ flux from ER to mitochondria through the IP_3_R and causes aggregation of α-syn in neurons (55), which is a hallmark of PD cytopathology.

Due to a low affinity of the MCU to Ca^2+^ ions, the local cytosolic Ca^2+^ concentration must reach ~5–10 μM in order for MCU to produce significant transport of Ca^2+^ into the mitochondria matrix (104). This may explain why up to 20% of the mitochondrial surface is situated in nanometer proximity to the ER (105–107).

An increased ER Ca^2+^ concentration, e.g., by an α-syn aggregate stimulated SERCA will favor the Ca^2+^ transport to mitochondria (Figure 1).

## Conclusion

Recent evidence points to a critical role for dysregulation of Ca^2+^ fluxes into the ER from the cytosol and from ER into mitochondria in the development of PD and other synucleinopathies. The evidence is based on genetic, biochemical and molecular studies. Promising preclinical data demonstrate disease modifying potential of targeting the SERCA pump, Ca^2+^ channels RyR, IP_3_R and IP_3_ regulating enzymes. This holds promise for identification of a new lines of disease modifying drug targets that can be tested in the near future.

## Author Contributions

All authors listed have made a substantial, direct and intellectual contribution to the work, and approved it for publication.

## Funding

The study was supported by Lundbeck Foundation grants R223-2015-4222 for PHJ, R248-2016-2518 for Danish Research Institute of Translational Neuroscience-DANDRITE, Nordic-EMBL Partnership for Molecular Medicine, Aarhus University, Denmark. LR has been supported by The Michael J Fox Foundation (grant ID 18283) and The EU Joint Programme-Neurodegenerative Disease Research (JPND) and The VELUX Foundations for the project OligoFIT. GK has been supported by the Novo Nordisk Foundation Distinguished Innovator 1-2020 award to Claus E. Olesen.

## Conflict of Interest

The authors declare that the research was conducted in the absence of any commercial or financial relationships that could be construed as a potential conflict of interest.

## Publisher's Note

All claims expressed in this article are solely those of the authors and do not necessarily represent those of their affiliated organizations, or those of the publisher, the editors and the reviewers. Any product that may be evaluated in this article, or claim that may be made by its manufacturer, is not guaranteed or endorsed by the publisher.
